# Cod-Liver Oil Substitute

**Published:** 1890-10

**Authors:** 


### COD-LIVER OIL SUBSTITUTE.

A physician writes : "It cannot be too widely known that cream separated by machinery (centrifugal cream separator) from pure new milk, before it has cooled, is a full substitute for cod-liver oil. If, during cold weather, those with a delicate constitution needing concentrated nutrition, but unable to overcome the nausea associated with cod-liver oil, will take this description of cream instead, they will find in most cases immense and lasting benefit. In several hospitals it has already quite suspended the nauseous oil."

In many cases milk does not agree with patients even when sipped slowly and used in small quantities. If there is no irritant or indigestible substance in the stomach, in most cases I have found new warm milk from a healthy cow, when slowly sipped, to be free from this objection and capable of more thorough and rapid digestion. After milk has stood five hours and over, it should, before being used as a beverage, be thoroughly shaken at least five minutes in a large bottle or an apparatus used for making single glasses of lemonade (simply a tin funnel fitting tightly over an ordinary drinking glass or goblet).

Violently agitating the milk in this manner recombines its various ingredients and makes it much easier of digestion. The ordinary "milk shake" is not such an unphilosophical drink after all, if the milk is pure. The home-made article, however, minus nauseous "pure fruit juices," for flavoring, is preferable, and judiciously used, cream and all will form an excellent food and substitute for cod-liver oils and preparations.

